# Life Cycle Assessment of Hybrid Nanofiltration Desalination Plants in the Persian Gulf

**DOI:** 10.3390/membranes12050467

**Published:** 2022-04-26

**Authors:** Benyamin Bordbar, Arash Khosravi, Ali Ahmadi Orkomi, Mohammad Peydayesh

**Affiliations:** 1 Sustainable Membrane Technology Research Group (SMTRG), Faculty of Petroleum, Gas and Petrochemical Engineering (FPGPE), Persian Gulf University (PGU), Bushehr P.O. Box 75169-13817, Iran; benyaminbordbar@gmail.com; 2 Department of Environmental Sciences, Faculty of Natural Resources, University of Guilan, Sowmeh Sara P.O. Box 43619-96196, Iran; orkomi@guilan.ac.ir; 3 Department of Health Sciences and Technology, ETH Zurich, 8092 Zurich, Switzerland

**Keywords:** life cycle assessment (LCA), hybrid desalination, multi-stage flash (MSF), reverse osmosis (RO), nanofiltration (NF)

## Abstract

Although emerging desalination technologies such as hybrid technologies are required to tackle water scarcity, the impacts of their application on the environment, resources, and human health, as prominent pillars of sustainability, should be evaluated in parallel. In the present study, the environmental footprint of five desalination plants, including multi-stage flash (MSF), hybrid reverse osmosis (RO)–MSF, hybrid nanofiltration (NF)–MSF, RO, and hybrid NF–RO, in the Persian Gulf region, have been analyzed using life cycle assessment (LCA) as an effective tool for policy making and opting sustainable technologies. The comparison was based on the impacts on climate change, ozone depletion, fossil depletion, human toxicity, and marine eutrophication. The LCA results revealed the superiority of the hybrid NF–RO plant in having the lowest environmental impact, although the RO process produces more desalinated water at the same feed and input flow rates. The hybrid NF–RO system achieves 1.74 kg CO_2_ equivalent, 1.24 × 10^−7^ kg CFC-11 equivalent, 1.28 × 10^−4^ kg nitrogenous compounds, 0.16 kg 1,4-DB equivalent, and 0.56 kg oil equivalent in the mentioned impact indicators, which are 7.9 to 22.2% lower than the single-pass RO case. Furthermore, the sensitivity analysis showed the reliability of the results, which helps to provide an insight into the life cycle impacts of the desalination plants.

## 1. Introduction

Global warming is one of the major conflicts in today’s world, and freshwater supply is a prominent challenge of sustainability. Water shortage has increased due to global warming, population growth, industrialization, and pollution of freshwater resources due to anthropogenic activities. The world’s population is estimated to increase by more than two billion by the next three decades [1]. Currently, more than one billion people in the world live in water-scarce areas. Water consumption has increased more than fivefold in the last century [2]. Water scarcity is affected by the supply and demand cycle. It is predicted that the average renewable water in Persian Gulf region is about 1000 cubic meters per capita per year, while the global average is more than 5000 cubic meters per capita per year. Additionally, the capacity of common water resources is endangered by increasing water demand and declining surface and groundwater quality. In order to solve this shortage, the countries located in the Persian Gulf region commenced the implementation of seawater desalination plants; however, the environmental impacts of seawater desalination have not been fully considered in development policies [3].

Seawater desalination is performed by a variety of processes and technologies. In general, desalination technologies can be classified into three categories: thermal, chemical, and membrane-based technologies [4]. Thermal processes include methods that use thermal energy to separate impurities from water, such as multi-stage flash (MSF), multi-effect distillation (MED), and thermal vapor compression (TVC). These technologies have high costs in addition to high thermal energy consumption. However, they have been prevalent in the past and are still used today. MSF is the most common thermal process [3,5]. The MSF water treatment process contributes significantly to the global capacity of the installed treatment plants and was the most common treatment technology in the Middle East [6]. Chemical methods desalinate seawater using chemical processes such as ion exchange resins. In membrane methods, water is purified and desalinated using a membrane. Some of these methods include RO, NF, microfiltration (MF), ultrafiltration (UF), electrodialysis (ED), etc. [5]. Membrane technology currently has an essential role in treating and desalinating seawater, and more than 60% of purified water is obtained using membrane technologies [5].

Economic constraints and technical specifications including the capacity, accessible technologies and specification of the feed are key factors in selecting the appropriate technology. However, desalination plants significantly impact the environment and natural resources and have direct and indirect emissions of pollutants into water, soil, and air through energy and chemical consumption [6,7]. Although seawater desalination is a well-established technology in the region, the assessment of environmental hazards and damages has not yet been adequately and thoroughly reviewed and considered by governments and industries [7,8].

Hybrid technologies are a new approach, which gain the benefits of two or more technologies simultaneously [5]. However, new technologies pose new challenges to ecosystems, resources, and human health. As per the proposal of Kloepffer, LCA is one of life cycle sustainability assessment concepts in sustainability studies [9,10]. LCA is used to assess and calculate the damage caused to the environment by a product or a process, which can be examined with several approaches, such as cradle to grave, gate to gate, cradle to gate, etc. [6,7,10,11,12,13].

There are several studies worldwide which have evaluated various aspects of desalination, including some the effects on the environment and human life. At first, pollutants’ emissions of desalination were assessed qualitatively without LCA [7]. Basic LCA studies on desalination processes were started in the 1990s; however, in the 2000s, research processes improved, and new quantitative comparisons between desalination technologies were published by applying LCA [7]. In the early 2000s, Lundie et al. investigated the environmental impacts of the RO process [14]. Raluy et al. compared RO, MSF, and MED processes and integration with renewable energy resources [15,16,17,18,19]. At the same time, Stokes and Horvath analyzed environmental emissions of an RO plant in the United States [20]. Furthermore, they assessed the role of renewable energy on air pollution caused by water supply plants [21]. In 2008, two studies by Vince et al. investigated the midpoint impacts of several RO and UF processes located in France [7,22,23]. Muñoz et al. studied several life cycle impacts of RO processes in Spain, in four projects [7,24,25,26].

In the 2010s, more researchers around the world started investigating the environmental and economic life cycle impacts of desalination plants. Beery et al. assessed environmental emissions of several RO and hybrid RO–UF plants in Germany [7,27,28,29]. Analyzing midpoint and endpoint impacts of various RO configurations was the most common topic of LCA studies in the 2010s and 2020s [7]. Some studies focused only on the environmental impacts of operating RO plants [30,31,32,33,34,35]. Moreover, some analyzed and compared RO with other traditional processes [36,37,38,39,40]. Recently, more studies focused on investigating and comparing emerging technologies [5,7]. Hancock et al., Al-Sarkal and Arafat, and Linares et al., studied the environmental impacts of hybrid technologies and compared them with individual processes [41,42,43]. Furthermore, Antipova et al. and Cherif et al. assessed the role of renewable energy in desalination plants [44,45]. In addition, several researchers analyzed both environmental and economic impacts [46,47,48,49]. On the other hand, it should be considered that the sustainability studies might be somewhat region-based because the sources of energy and available technologies in the regions are different. For example, the impacts of using an electricity grid in the Middle East and the Europe are totally different due to different sources of power production in addition to the available capacity of renewable energies in these regions [7].

**Figure 1:**
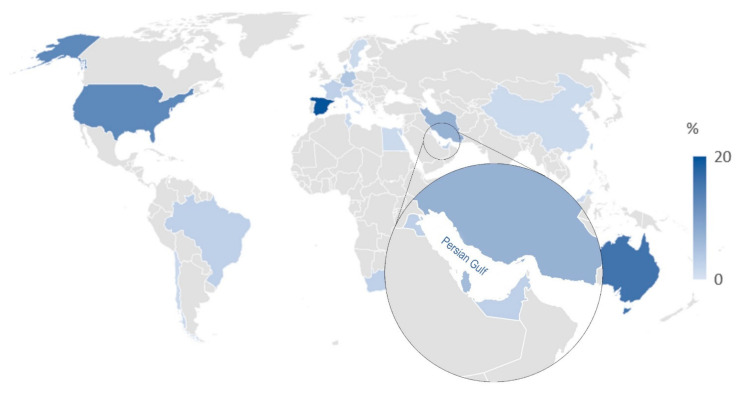
Distribution of water desalination LCA studies worldwide [7].

Recent literature in nanofiltration desalination plants focuses on the emerging technologies’ environmental impact, such as hybrid processes, zero liquid discharge (ZLD) technologies, and the effect of renewable energy usage [5,7]. Ronquim et al. analyzed and compared the midpoint impacts of global warming, energy resources depletion, land use, and mineral resources depletion indicators for RO and ZLD processes [50]. Furthermore, Tsalidis et al. investigated ZLD plants in some European countries [51]. Recently, Khosravi et al. reviewed the LCA studies of emerging technologies in industrial wastewater treatment and desalination globally, and Figure 1 presented the distribution of LCA and sustainability studies of desalination plants in different territories and regions [7].

In this study, the LCA of five up-to-date real hybrid desalination plants in the Persian Gulf region, including recirculation multi-stage flash (R-MSF), hybrid RO/R-MSF, hybrid NF/R-MSF, single-pass RO, and hybrid NF/RO with a cradle-to-gate approach are assessed, and their emissions and environmental impacts are calculated, reviewed and compared. The contribution of the effective parameters (such as electricity, thermal energy, chemicals, and materials) on the impacts is also described. This study investigated the midpoint environmental impacts of emerging technologies from a long-term point of view and compared them with the traditional technologies, which helps to improve deep-seated plans to reduce their environmental impact and determine a policy for applying desalination technologies in the region.

## 2. Methodology

### 2.1. Goals and Scopes

LCA was implemented to investigate the life cycle impacts of five hybrid desalination plants. Case 1 is an R-MSF plant; case 2 is a hybrid RO/R-MSF plant; case 3 is a hybrid NF/R-MSF plant, case 4 is an RO plant, and case 5 is a hybrid NF/RO plant. The R-MSF desalination plant (case 1) is a real plant in operation in the Persian Gulf region as described and studied by Mannan et al. [8]. Case 2 is a pilot case where the R-MSF process is combined with RO (RO/R-MSF). In case 3, a pilot hybrid of R-MSF and NF technology (NF/R-MSF) is being conducted [8]. Case 4 is a single-stage RO plant, and case 5 is a hybrid NF/RO plant. Materials inventory, energy consumption, and membrane modules information have been extracted and used from industrial sources [8,52,53,54,55,56].

The cradle-to-gate approach of LCA is used to investigate the environmental impacts of the energy, chemicals, and materials used in the production of membranes and other parts of the aforementioned desalination plants, in addition to the impacts of energy and chemical consumption during the operation. SimaPro 9.3 software, Ecoinvent 3.8 cut-off database, and ReCipe 1.13 midpoint [57] method with global characterization factors were used to analyze the long-term impacts of the aforementioned cases.

### 2.2. Systems and Functional Unit

**Figure 2:**
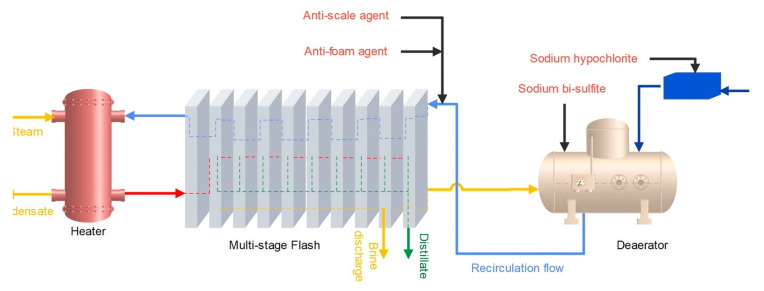
The schematic of the R-MSF system.

R-MSF was once the most common desalination method in Persian Gulf region. In the MSF process, seawater feed passes through pipes that are heated by the thermal energy of steam. The steam is in contact with the incoming saltwater pipe, leading to water evaporation [4]. In the chambers, the pressure decreases gradually in each stage, compared with the previous stage. When the heated seawater enters a low-pressure chamber, it suddenly evaporates. The vapor condenses by heat exchanging via the feed tubes on the top of the chamber and the condensate is collected by a vessel inside the chamber. The remaining saline water, called brine, exits the bottom of the chamber and re-enters the process in a cycle. High concentration brine is discharged into the sea. The inlet water pressure is less than 3 bar and the temperature is about 100 °C [8,58]. In addition, sodium hypochlorite is added to control biological growth in the desalination plant (chlorination process), and sodium bisulfite is added to control corrosion by removing dissolved gases (deaeration). The functional unit for LCA is 1 m^3^ of produced freshwater. The schematic of case 1 (R-MSF) is depicted in Figure 2.

The required steam in case 1 is obtained from the steam returned from the turbine of a natural gas combined cycle power plant. This is undertaken to reduce energy consumption. The required thermal energy in the studied cases (cases 1–3) are 107, 64, and 64 MJ/m^3^, respectively [8]. Cases 4 and 5 do not require any thermal energy.

**Figure 3:**
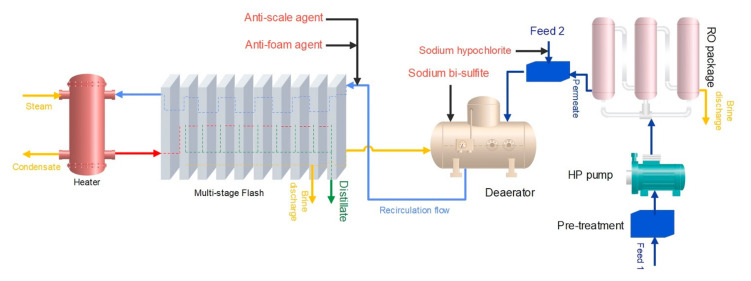
The schematic of case 1 (hybrid RO/R-MSF Flash).

The feed temperature in case 1 is kept below 112 °C to restrict the formation of scale due to the presence of calcium carbonate, calcium sulfate, and magnesium hydroxide in the feed. By adding RO and NF processes before the MSF process, these substances are reduced in the feed of MSF, and the MSF inlet temperature can be raised, which will ultimately increases plant productivity [8]. The schematic of the hybrid RO/R-MSF system is shown in Figure 3.

**Figure 4:**
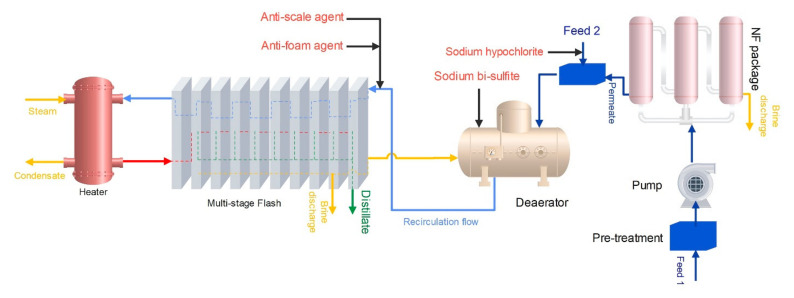
The schematic of case 3 (hybrid NF/R-MSF).

Figure 4 shows the schematic of case 3 (hybrid NF/R-MSF). As shown in Figure 3 and Figure 4, half the feed enters the membrane process, and the other half is mixed with permeate flow and enters MSF.

**Figure 5:**
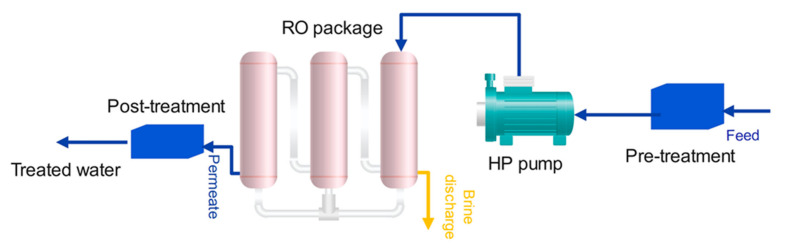
The schematic of the Reverse Osmosis system.

Case 4 is a single-stage RO plant. In this process, the seawater feed enters the RO system. At first, a pre-treatment process applied in order to remove substances that may cause membrane fouling, scaling and corrosion. Then the feed enters a high-pressure pump. The pump increases the pressure up to 16 bar to supply the transmembrane pressure (TMP) required for RO membrane modules. The required electrical energy for this process is 4.22 kWh for 1 cubic meter of desalted water [59]. The schematic of the RO plant is illustrated in Figure 5.

**Figure 6:**
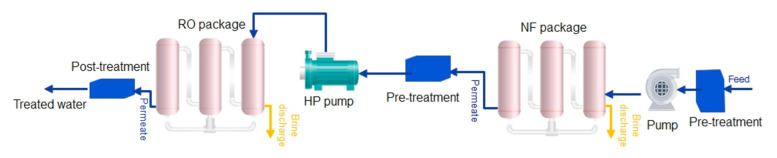
The schematic of case 5 (hybrid NF–RO).

The last case is a hybrid NF–RO plant. After pre-treatment, the seawater feed enters a pump to supply the required TMP for NF membrane modules, then the feed enters the NF modules. The permeate goes to the high-pressure pumps to be treated by the RO process. Due to the pumping system at the NF process, the NF permeate pressure is more than the feed of RO in case 4; therefore, less electricity is needed for RO high-pressure pumps. The required electricity of this plant is 3.11 kWh for 1 cubic meter of desalted water [59]. The schematic of the hybrid NF–RO plant is drawn in Figure 6.

The chemical additives used in pre-treatment and post-treatment of cases 1–5 are the same, except more chemicals are added to the feed to prevent fouling and corrosion of membranes in plants 2–5. Additionally, due to the high sensitivity of the RO membrane, additive chemicals such as citric acid and sodium sulfite are added exclusively to cases 2, 4 and 5. High-temperature antifouling is used in all cases to prevent the formation of scale due to the presence of calcium carbonate, calcium sulfate, and magnesium hydroxide in the feed at high temperatures. Applying high temperature increases the MSF performance. Other common pre-treatment methods include aeration and chlorination, the addition of sodium hydrogen sulfate, and ethanol to control descaling and foaming. To maintain distilled quality and control the growth of aquatic organisms, treatment in all plants is performed using calcium hydroxide and chlorination demineralizing agents. Chlorination, coagulation, and removal of the medium followed by the cartridge is used as a pre-treatment method for the NF system. In addition to common additives, citric acid and sodium sulfate are injected in the pre-treatment of RO in order to avoid membrane fouling and scaling, and hydrated lime and carbon dioxide are added to demineralize the water at the post-treatment stage [52,53]. Additionally, the environmental effects of the materials and solvents used for the membrane modules synthesis have been considered.

### 2.3. Life Cycle Inventory Analysis

In the life cycle inventory (LCI) stage, the data and information needed to estimate the emission rates of each process were collected [7]. In this study, the required data for cases 1 to 5 were collected from industrial data [8,52,53,54,55,56]. Table 1 shows the information for cases 1 to 5. More details about the data collection phase are presented in the supplementary section (Tables S1 to S5).

Some chemicals are used in pre-treatment and post-treatment of the processes. Sodium hypochlorite is added to control microorganisms, bacteria, and other biological factors. To prevent corrosion, sodium bisulfite is used as a pre-treatment. Scaling is a challenging factor for desalination plants, where anticalins such as sulfuric acid and anti-foaming objects such as monoethylene oxide are added. The coagulant, iron chloride, is added to the NF and RO pre-treatment; however, in the post-treatment, sodium hypochlorite is added to the NF and RO process. Carbon dioxide and sodium hydroxide are used for the post-treatment of RO, and sodium sulfite is added as pre-treatment. The values of the additive chemicals are the dosages in water stream during the treatment, which are extracted from the industrial data [8,52,53] (Table 2).

Components such as polyester, polysulfone, *N*,*N*-dimethylformamide (DMF), meta-phenylene diamine (MPD), trimesoyl chloride (TMC), isopropanol (IPA), and phosphoric acid are the materials and solvents used to synthesize the membrane layer; and polypropylene as spacer, epoxy resin as glue and PVC as permeate tube are used. The data relating to the NF module were collected from the specifications of the commercial 8-inch NF membrane [6] and are listed in Table 3. The membrane lifetime was considered to be 4 years.

As for the NF modules, the environmental effects in the production phase of the RO modules were also considered. The data of the RO module were collected from SimaPro9.3 software database related to an 8-inch operating module [6]. The lifetime of membrane modules was considered to be 4 years. The inventory data of the RO module are been presented in Table 4.

### 2.4. Life Cycle Impact Assessment (LCIA)

In the life cycle impact assessment (LCIA) phase, the environmental impacts of each process were calculated by using the emission inventories and the environmental impact potential of emitted materials [7]. The mentioned desalination processes release abundant pollutants and cause numerous environmental issues over their life cycle. The most common are CO_2_, SO_X_, NO_X_, and different sized dust particles. Another important issue is fossil energy resources consumption [60]. The *ReCipe* method can assess 18 types of midpoint impacts; only 5 (climate change, ozone depletion, marine eutrophication, human toxicity, and fossil depletion) were examined in this study, in order to distinguish the cases regarding their environmental impacts. Climate change (CC) indicates the emission of equivalent carbon dioxide. Ozone depletion potential (ODP) represents the amount of CFC-11 equivalent released. Marine eutrophication potential (MEP) reveals the impacts of nitrogenous and phosphorous compounds. Human toxicity potential (HTP) indicates the degree of toxicity to humans, and fossil depletion potential (FDP) is also calculated based on consumed oil [7].

## 3. Results and Discussion

### 3.1. Life Cycle Impact Assessment (LCIA)

**Figure 7:**
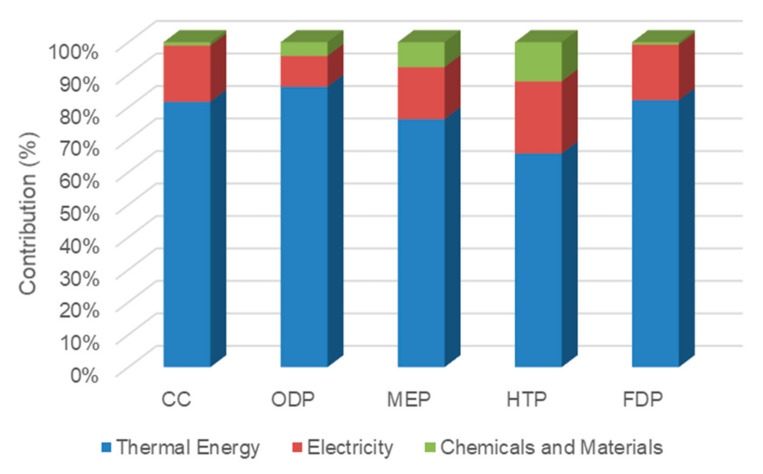
The contribution of inventories in midpoint impacts of case 1.

As shown in Figure 7, thermal energy and electricity consumption in case 1 had the largest contribution in all impacts. The thermal energy and electricity contributed to about 81.7% and 17.3% of CC index, respectively. This is due to the greenhouse gas emission from natural gas combustion in the gas boiler for steam and electricity production in a combined cycle power plant. Furthermore, chemicals and materials were responsible for 1.04%, 4.23%, 7.66%, 12.1%, and 0.749% share in CC, ODP, MEP, HTP and FDP indicators, respectively.

**Figure 8:**
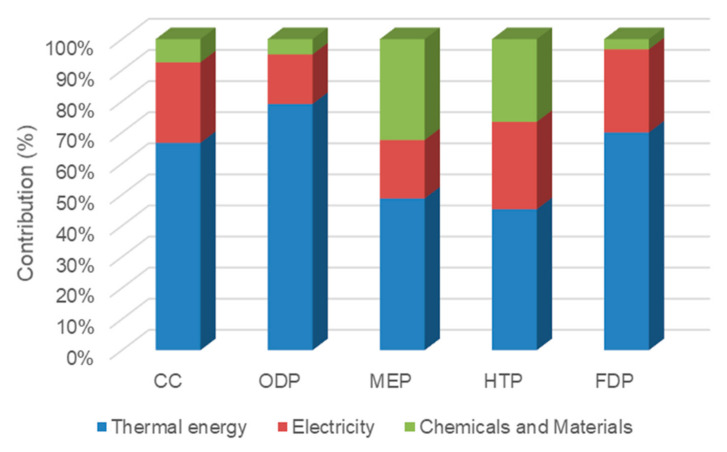
The contribution of inventories in midpoint impacts of case 2.

As shown in Figure 8, the share of electrical energy impacts in case 2 increased by comparison with case 1 because more electrical energy is consumed in high-pressure pumps of the RO process. The electrical energy contributions in the five mentioned impacts were 25.9%, 15.9%, 18.8%, 28.1%, and 26.7%, respectively. The thermal energy impact was still the highest in all indicators. In this case, the effect of chemicals and materials on MEP was higher than electricity, which contributed to a share of 32.4%, due to materials consumed for membrane fabrication and module production. In raw chemical and material production, membrane fabrication, and module packaging processes, a large amount of chemicals and materials are consumed or emitted, leading to changing the level of eutrophication.

**Figure 9:**
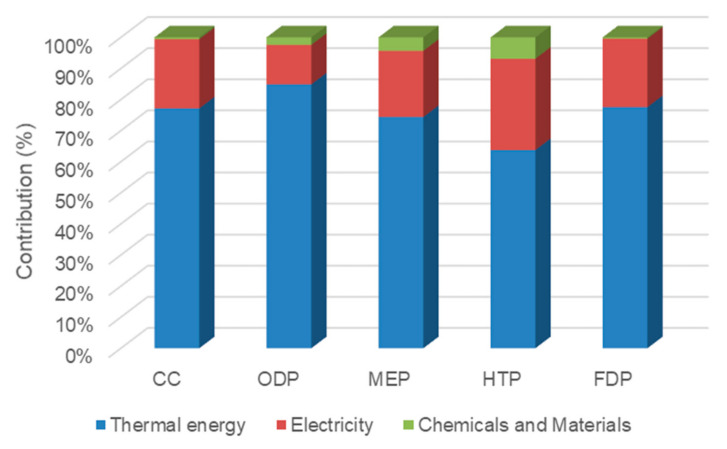
The contribution of inventories in midpoint impacts of case 3.

In case 3, the electricity consumption was lower than case 2. However, the consumption of thermal energy was as same as case 2. The relative contribution of thermal energy in midpoint impact indicators of case 3 (Figure 9) was higher compared with case 2 (Figure 8) due to lower electricity, chemicals, and materials consumptions. The contributions of thermal energy for the five mentioned impacts were 77.2%, 84.9%, 74.4%, 63.8% and 77.6%, respectively.

**Figure 10:**
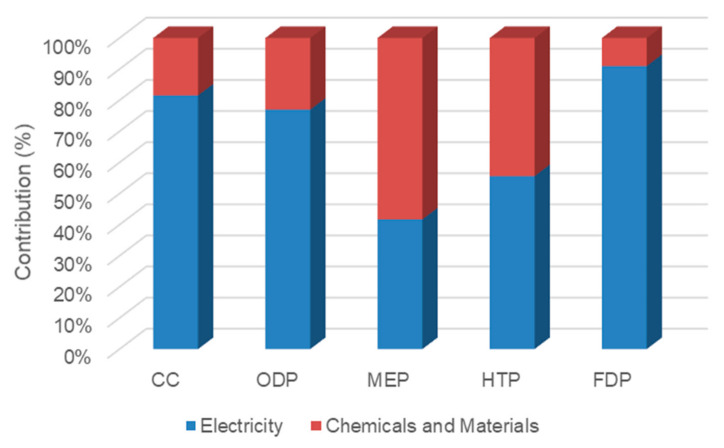
The contribution of inventories in midpoint impacts of case 4.

In case 4 (Figure 10), a single-pass RO process operates to desalinate feed seawater. In this case, thermal energy is not used; however, more electrical energy is applied for high-pressure pumps, the whole feed seawater enters the RO process, and the flowrate of pumps is twice than that of case 2. Electricity contributed to 81.6%, 77%, 41.7%, 55.6%, and 91% of CC, ODP, MEP, HTP, and FDP indices, respectively. Due to the absence of thermal energy, the operational and construction materials and chemicals had a major effect on the MEP indicator (58.3%).

**Figure 11:**
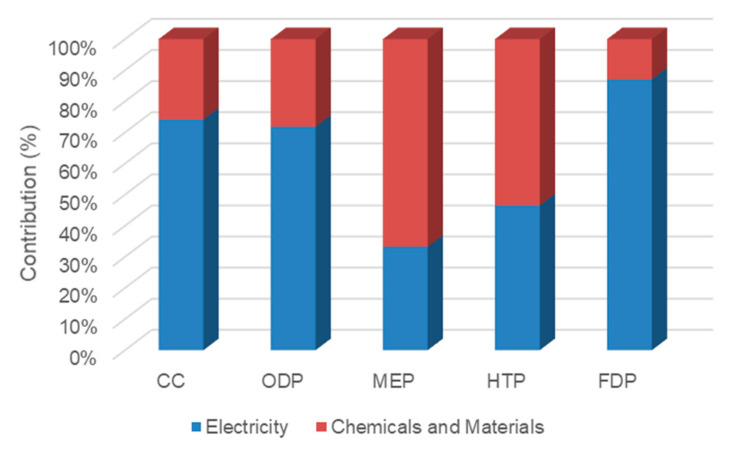
The contribution of inventories in midpoint impacts of case 5.

Figure 11 represents the contribution inventories in each midpoint impact in case 5. This case consists of a single-pass NF and single-pass RO in series. Due to the pressure applied by the pump before the NF process, less load is needed for the high-pressure pump; therefore, electricity consumption is less than that of case 4. On the other hand, more membrane modules are used due to applying the NF process, and more materials were manipulated rather than in a single RO case. The share of electricity was 74.2%, 71.9%, 33.3%, 46.6%, and 87.1% for the mentioned midpoint indicators, respectively. Moreover, MEP and HTP indicators were affected by chemicals and materials more than electricity.

**Figure 12:**
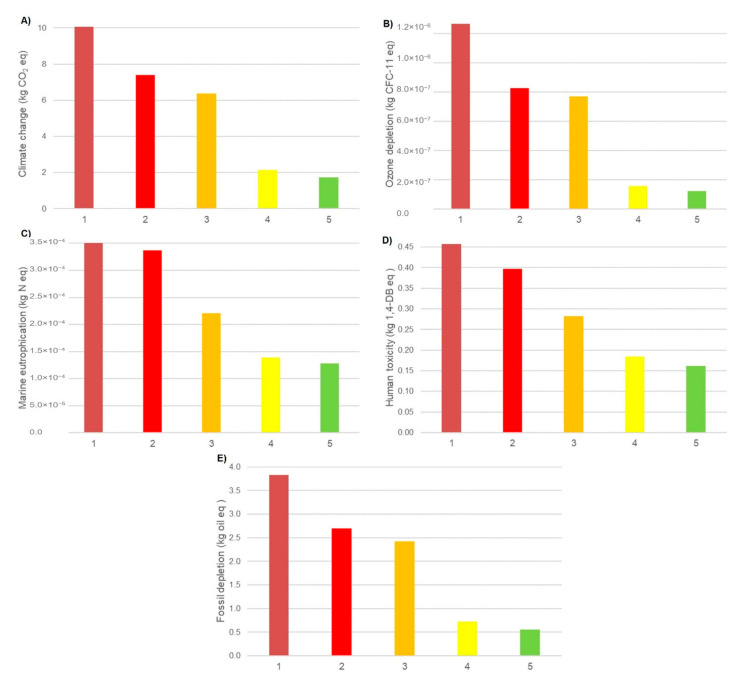
The impacts of desalination plants of cases 1 to 5 on (**A**) climate change, (**B**) ozone depletion, (**C**) marine eutrophication, (**D**) human toxicity, and (**E**) fossil depletion.

Figure 12 shows a comparison between the cases regarding the indicators. The analysis of the midpoint environmental factors for cases 1–5 revealed that case 1 (MSF) had the most impact on all five factors, which shows that the MSF process individually has the most environmental impact and emissions. The emission rate of combustion-induced gases such as CO_2_, SO_X_, and NO_X_ in case 1 was more than in other cases due to burning a large amount of natural gas to provide thermal energy. Case 2 (RO/R-MSF) was in the second place, due to lower thermal energy consumption compared with case 1. As a result, its environmental footprint was less than case 1. However, the high mechanical energy used in the high-pressure pumps caused high environmental impact.

Case 3 showed lower risks due to lower thermal energy usage than case 1 and lower electricity consumption than case 2. No thermal energy was applied in cases 4 and 5; therefore, they emitted less waste than other cases. The RO case (case 4) consumed more electrical energy than the hybrid NF/RO case (case 5) because of the greater load applied in the high-pressure pump. The last case achieved the lowest values in all the midpoint indicators, indicating than case 5 was superior to the other four cases regarding environmental footprint. It is noteworthy that the efficiency of case 5 was less than cases 2 and 4, and that the NF product quality cannot be as high as the RO technology. The RO system is able to treat 60% of the feed, versus 45% for the hybrid NF–RO process. The environmental impacts of all five conducted cases are shown in Table 5.

### 3.2. Sensitivity Analysis

Risk assessment is a scientific procedure to describe and determine the uncertainty and its characterizations to define or change decisions. A set of systematic, logical, analytical, evidence-based procedures were performed to find and measure the risks, probability, and possibility of a process and a way for decision-making [61]. Sensitivity analysis helps to improve the design and model by assessing the qualitative and quantitative responses of the studied analysis [62].

**Figure 13:**
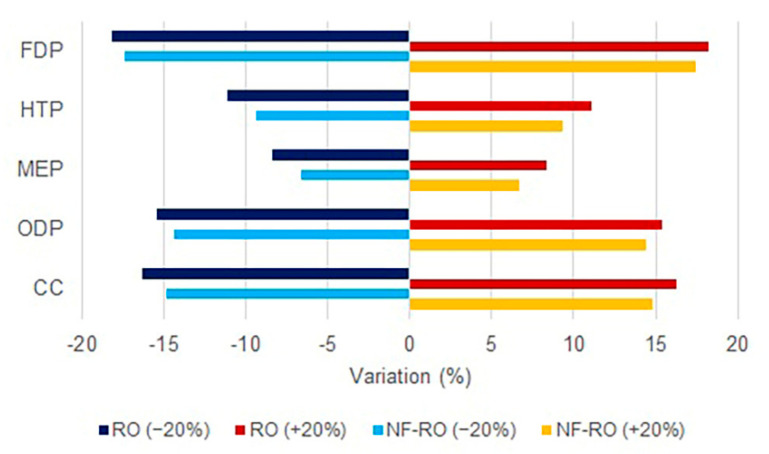
Sensitivity analysis for cases 4 and 5 based on the impacts of the electricity variation.

Due to the significant environmental impacts of electricity compared with the other inventories in the five studied cases, the sensitivity analysis was applied to the electrical energy with ±20% variation for cases 4 and 5. Electricity is the most effective parameter affecting CC, ODP, and FDP impact indicators due to burning natural gas in combined cycle power plants to supply electricity for mechanical equipment. The sensitivity analysis results showed that by 20% variation of electricity in case 4, the CC, ODP, MEP, HTP, and FDP indicators were varied at 16.31%, 15.4%, 8.34%, 11.12%, and 18.2%, respectively. Moreover, for the last case (hybrid NF–RO), the mentioned indicators were varied by 14.84%, 14.38%, 6.67%, 9.32%, and 17.42%, respectively (Figure 13). Generally, sensitivity analysis found that by ±20% changes in electrical energy, the environmental impacts changed from 8.34% to 18.2% for RO, and 6.67% to 17.42% for hybrid NF–RO processes by applying more efficient equipment or choosing more sustainable energy resources.

## 4. Conclusions

In this study, five hybrid desalination plants, including R-MSF, RO/R-MSF, NF/R-MSF, RO, and NF/RO in the Persian Gulf region, were examined by the LCA method. The results of this study may be helpful to reduce environmental impacts and determine a sustainable policy for developing desalination projects, considering the vital need for water resources and the growing development of seawater desalination technologies. There is a high potential to reduce the environmental impact of MSF desalination by increasing the efficiency of the process via flash stage addition or advanced feed water pre-treatment using NF or RO. Furthermore, membrane-based technologies such as NF and RO may be considered a good alternative for traditional processes such as thermal processes, which do not need thermal energy. This study revealed that by applying RO and hybrid NF/RO technologies, a notable reduction in environmental impact indicators might be expected compared with traditional technologies. The results showed that the hybrid NF/RO technology had the minimum environmental impact, which could guide the design of new plants in the future. However, it should be noted that the RO system can treat 60% of feed water, versus 45% for the hybrid NF/RO system, as studied in this work. The sensitivity analysis determined how the quantity of electrical energy would change the environmental impacts. It found that by ±20% changes in electrical energy, the impact indictors may change from 8.34% to 18.2% for RO, and from 6.67% to 17.42% for hybrid NF–RO processes, which means that by applying more efficient equipment or choosing clean energy resources, the environmental footprint can be reduced.

## Figures and Tables

**Table 1:** Plant configuration and energy demand of the cases 1–5.

Plant Specification	Case 1	Case 2	Case 3	Case 4	Case 5
Technology	R-MSF	Hybrid RO/R-MSF	Hybrid NF/R-MSF	RO	Hybrid NF/RO
Configuration	Cross tube MSF	Cross tube MSF Single-pass RO	Cross tube MSF Single-pass NF	Single-pass RO	Single-pass NF Single-pass RO
Number of stages	21	35	35	-	-
Thermal energy (MJ)	107	64	64	-	-
Electrical energy (kWh/m^3^)	4.19	4.6	3.42	4.22	3.11
Feed (seawater) Flowrate (m^3^/h)	28,000	675	675	675	675
Treated water flowrate (m^3^/h)	3430.57	378	288.1	405	303.75
Reference	[8]	[52,56]	[8]	[52,56]	[52,55,56]

**Table 2:** Chemical additives in pre-treatment and post-treatment of plants.

Stage	Chemicals	Amount (ppm)
Pre-treatment cases 1–5	Sodium hypochlorite	4
	Sodium bisulfite	0.5
	Sulfuric acid	2.4
	Monoethyleneoxide	0.1
Post-treatment for cases 1–5	Calcium hydroxide	0.5
	Sodium hypochlorite	0.5
Pre-treatment for NF and RO system	Ferric chloride	0.3
Post-treatment for NF and RO system	Chlorine	0.2
Pre-treatment for RO system	Citric acid	0.937
	Sodium sulfite	0.0739
Post-treatment for RO system	Lime	51.03
	Carbon dioxide	43

**Table 3:** Materials usage in fabrication of NF modules for their lifetime.

Component	Amount (kg/m^3^)
Polyester	4.79452 × 10^−11^
Polysulfone	1.0274 × 10^−11^
DMF (*N*,*N*-dimethylformamide)	4.10959 × 10^−11^
MPD (meta-phenylene diamine)	4.62329 × 10^−13^
TMC (trimesoyl chloride)	1.19178 × 10^−12^
Phosphoric acid	3.20548 × 10^−12^
Polypropylene (spacers)	5.13699 × 10^−11^
Epoxy resin (glue)	1.16438 × 10^−11^
PVC (permeate tube)	1.78082 × 10^−11^
IPA (isopropanol)	5.82192 × 10^−12^

**Table 4:** Materials usage in fabrication of RO modules for their lifetime.

Component	Amount (kg/m^3^)
ABS (Acrylonitrile-butadiene-styrene copolymer)	2.90964 × 10^−^^13^
Polyester	2.0077 × 10^−13^
Polysulfone	2.12833 × 10^−13^
DMF (*N*,*N*-dimethylformamide)	8.52231 × 10^−12^
MPD (meta-phenylene diamine)	9.00922 × 10^−16^
TMC (trimesoyl chloride)	2.69506 × 10^−15^
Phosphoric acid	6.31159 × 10^−14^
Polypropylene (spacers)	4.26565 × 10^−13^
Epoxy resin (glue)	1.17299 × 10^−14^
PVC (permeate tube)	1.3347 × 10^−15^
IPA (isopropanol)	2.10215 × 10^−14^

**Table 5:** Midpoint environmental indicator values for cases 1–5.

Impact	Unit	Case 1	Case 2	Case 3	Case 4	Case 5
Climate change	kg CO_2_ eq	10.08	7.39	6.38	2.15	1.74
Ozone depletion	kg CFC-11 eq	1.27 × 10^−6^	8.26 × 10^−7^	7.70 × 10^−7^	1.57 × 10^−7^	1.24 × 10^−7^
Marine eutrophication	kg N eq	3.60 × 10^−4^	3.37 × 10^−4^	2.21 × 10^−4^	1.39 × 10^−4^	1.28 × 10^−4^
Human toxicity	kg 1,4-DB eq	0.46	0.40	0.28	0.18	0.16
Fossil depletion	kg oil eq	3.83	2.69	2.43	0.72	0.56

## Data Availability

Not applicable.

## Supplementary Materials

The following supporting information can be downloaded at: https://www.mdpi.com/article/10.3390/membranes12050467/s1, Table S1: Life cycle inventory of case 1, Table S2: Life cycle inventory of case 2, Table S3: Life cycle inventory of case 3, Table S4: Life cycle inventory of case 4, Table S5: Life cycle inventory of case 5.
