# Supplementary material for: Life Cycle Assessment of Hybrid Nanofiltration Desalination Plants in the Persian Gulf
Source: Membranes (Basel). 2022 Apr 26;12(5):467. doi: 10.3390/membranes12050467 (PMC9147468; doi:10.3390/membranes12050467)
Supplement: Supplementary file 1 [file membranes-12-00467-s001.zip › membranes-1682776-supplementary.pdf]

membranes

Supplementary file for

Life Cycle Assessment of Hybrid Nanofiltration Desalination Plants in the Persian Gulf

Benyamin Bordbar [1], Arash Khosravi [1,*], Ali Ahmadi Orkomi [2] and Mohammad Peydayesh [3,*]

[1] Sustainable Membrane Technology Research Group (SMTRG), Faculty of Petroleum, Gas and Petrochemical Engineering (FPGPE), Persian Gulf University (PGU), Bushehr P.O. Box 75169-13817, Iran; benyaminbordbar@gmail.com
[2] Department of Environmental Sciences, Faculty of Natural Resources, University of Guilan, Sowmeh Sara P.O. Box 43619-96196, Iran; orkomi@guilan.ac.ir
[3] Department of Health Sciences and Technology, ETH Zurich, 8092 Zurich, Switzerland
* Correspondence: arash.khosravi@pgu.ac.ir (A.K.); mohammad.peydayesh@hest.ethz.ch (M.P.)

Tables number : 5

Citation: Bordbar, B.; Khosravi, A.; Ahmadi Orkomi, A.; Peydayesh, M. Life Cycle Assessment of Hybrid Nanofiltration Desalination Plants in the Persian Gulf. *Membranes* **2022**, *12*, 467. https://doi.org/10.3390/membranes12050467

Academic Editor: Pei Sean Goh

Received: 30 March 2022
Accepted: 24 April 2022
Published: 26 April 2022

Publisher's Note: MDPI stays neutral with regard to jurisdictional claims in published maps and institutional affiliations.

Copyright: © 2022 by the authors. Licensee MDPI, Basel, Switzerland. This article is an open access article distributed under the terms and conditions of the Creative Commons Attribution (CC BY) license (https://creativecommons.org/licenses/by/4.0/).

Table S1. Life cycle inventory of case 1.

Materials/fuels	Amount	Unit
Sodium hypochlorite	0.004	kg
Sodium hydrogen sulfite	0.0005	kg
Sulfuric acid	0.0024	kg
Ethylene oxide	0.0001	kg
Lime	0.0005	kg
Sodium hypochlorite	0.0005	kg
Electricity	0.513360296	kWh
Heat	13.10967821	MJ

Table S2. Life cycle inventory of case 2.

Materials/fuels	Amount	Unit
Sodium hypochlorite	0.005	kg
Sodium hydrogen sulfite	0.000625	kg
Sulfuric acid	0.003	kg
Ethylene oxide	0.000125	kg
Lime	0.000625	kg
Sodium hypochlorite	0.000625	kg
Iron (III) chloride	0.0006	kg
Chlorine, liquid	0.0004	kg
Citric acid	0.001874	kg
Sodium sulfite	0.0001478	kg
Lime	0.1701	kg
Carbon dioxide	0.143333333	kg
Acrylonitrile-butadiene-styrene copolymer	2.90964×10^{-13}	kg
Polyester	2.0077×10^{-13}	kg
Polysulfone	2.12833×10^{-13}	kg
N,N-dimethylformamide	8.52231×10^{-12}	kg
Meta-phenylene diamine	9.00922×10^{-16}	kg
Trimesoyl chloride	2.69506×10^{-15}	kg
Phosphoric acid	6.31159×10^{-14}	kg
Polypropylene	4.26565×10^{-13}	kg
Epoxy resin	1.17299×10^{-14}	kg
Polyvinylchloride	1.3347×10^{-15}	kg
Isopropanol	2.10215×10^{-14}	kg
Electricity	2.53	kWh
Heat	35.2	MJ

Table S3. Life cycle inventory of case 3.

Materials/fuels	Amount	Unit
Sodium hypochlorite	0.004571429	kg
Sodium hydrogen sulfite	0.000571429	kg
Sulfuric acid	0.002742857	kg
Ethylene oxide	0.000114286	kg
Lime	0.000571429	kg
Sodium hypochlorite	0.000571429	kg
Iron (III) chloride	0.0006	kg
Chlorine	0.0004	kg
Polyester	4.79452×10^{-11}	kg
Polysulfone	1.0274×10^{-11}	kg
N,N-dimethylformamide	4.10959×10^{-11}	kg
Meta-phenylene diamine	4.62329×10^{-13}	kg
Trimesoyl chloride	1.19178×10^{-12}	kg
Phosphoric acid	3.20548×10^{-12}	kg
Polypropylene	5.13699×10^{-11}	kg
Epoxy resin	1.16438×10^{-11}	kg
Polyvinylchloride	1.78082×10^{-11}	kg
Isopropanol	5.82192×10^{-12}	kg
Electricity	1.459656	kWh
Heat	27.3152	MJ

Table S4. Life cycle inventory of case 4.

Materials/fuels	Amount	Unit
Sodium hypochlorite	0.006666667	kg
Sodium hydrogen sulfite	0.000833333	kg
Sulfuric acid	0.004	kg
Ethylene oxide	0.000166667	kg
Lime	0.000833333	kg
Sodium hypochlorite	0.000833333	kg
Iron (III) chloride	0.0005	kg
Chlorine	0.000333333	kg
Citric acid	0.001561667	kg
Sodium sulfite	0.000123167	kg
Lime	0.127575	kg
Carbon dioxide	0.1075	kg
Acrylonitrile-butadiene-styrene copolymer	2.90964×10^{-13}	kg
Polyester	2.0077×10^{-13}	kg
Polysulfone	2.12833×10^{-13}	kg
N,N-dimethylformamide	8.52231×10^{-12}	kg
Meta-phenylene diamine	9.00922×10^{-16}	kg
Trimesoyl chloride	2.69506×10^{-15}	kg
Phosphoric acid	6.31159×10^{-14}	kg
Polypropylene	4.26565×10^{-13}	kg
Epoxy resin	1.17299×10^{-14}	kg
Polyvinylchloride	1.3347×10^{-15}	kg
Isopropanol	2.10215×10^{-14}	kg
Electricity	2.532	kWh

Table S5. Life cycle inventory of case 5.

Materials/fuels	Amount	Unit
Sodium hypochlorite	0.004	kg
Sodium hydrogen sulfite	0.0005	kg
Sulfuric acid	0.0024	kg
Ethylene oxide	0.0001	kg
Lime	0.000666667	kg
Sodium hypochlorite	0.0005	kg
Iron (III) chloride	0.0003	kg
Chlorine	0.0002	kg
Citric acid	0.000937	kg
Sodium sulfite	0.0000739	kg
Lime	0.1134	kg
Carbon dioxide	0.095555556	kg
Acrylonitrile-butadiene-styrene copolymer	2.18223×10^{-13}	kg
Polyester	4.80958×10^{-11}	kg
Polysulfone	1.04336×10^{-11}	kg
N,N-dimethylformamide	4.74876×10^{-11}	kg
Meta-phenylene diamine	4.63005×10^{-13}	kg
Trimesoyl chloride	1.1938×10^{-12}	kg
Phosphoric acid	3.25282×10^{-12}	kg
Polypropylene	5.16898×10^{-11}	kg
Epoxy resin	1.16526×10^{-11}	kg
Polyvinylchloride	1.78092×10^{-11}	kg
Isopropanol	5.83769×10^{-12}	kg
Electricity	1.3995	kWh
